# Investigation of the Significance of Blood Signatures on Sepsis-Induced Acute Lung Injury in Sepsis Within 24 Hours

**DOI:** 10.1155/ijog/5684300

**Published:** 2025-05-19

**Authors:** Zaojun Fang, Yuanyuan Wang, Lingqi Xu, Ying Lin, Biao Zhang, Jiaping Chen

**Affiliations:** ^1^Suzhou TCM Hospital Affiliated to Nanjing University of Chinese Medicine, Suzhou, Jiangsu, China; ^2^Suzhou Research Center of Medical School, Suzhou Hospital, Affiliated Hospital of Medical School, Nanjing University, Suzhou, Jiangsu, China; ^3^Department of Emergency, Suzhou Hospital of Integrated Traditional Chinese and Western Medicine, Suzhou, Jiangsu, China

**Keywords:** ALI, machine-learning strategy, sepsis, *SLPI*

## Abstract

**Background:** Sepsis is an infection-induced dysregulated cellular response that leads to multiorgan dysfunction. As a time-sensitive condition, sepsis requires prompt diagnosis and standardized treatment. This study investigated the impact of biomarkers identified in peripheral whole blood from sepsis patients (24-h post-onset) on sepsis-induced acute lung injury (ALI) using bioinformatics and machine learning approaches.

**Methods:** Gene Expression Omnibus (GEO) datasets were analyzed for functional and differential gene expression. Critical genetic markers were identified and evaluated using multiple machine learning algorithms. Single-cell RNA sequencing (scRNA-seq) and cell-type identification by estimating relative subsets of RNA transcript (CIBERSORT) were conducted to explore associations between biomarkers and immune cells. Biomarker expression was further validated through animal experiments.

**Result:** A total of 611 overlapping differentially expressed genes (DEGs) were identified in GSE54514, including 361 upregulated and 250 downregulated genes. From GSE95233, 1150 DEGs were detected, with 703 upregulated and 447 downregulated genes. Enrichment analysis revealed DEGs associated with immune cell activity, immune cell activation, and inflammatory signaling pathways. Component 3a receptor 1 (C3AR1) and secretory leukocyte peptidase inhibitor (SLPI) were identified as critical biomarkers through multiple machine learning approaches. CIBERSORT analysis revealed significant associations between immune cell types and C3AR1/SLPI. Moreover, the scRNA-seq analysis demonstrated that the SLPI expression was significantly elevated in immunological organ cells during the early stages of sepsis, a finding further validated in sepsis-induced ALI models.

**Conclusion:** This study employed machine learning techniques to identify sepsis-associated genes and confirmed the importance of SLPI as a biomarker within 24 h of sepsis onset. SLPI also played a significant role in sepsis-induced ALI, suggesting its potential as a novel target for personalized medical interventions, targeted prevention, and patient screening.

## 1. Introduction

Sepsis, a life-threatening disease, remains the leading cause of in-hospital mortality [1]. Organ dysfunction, septic shock, and related complications are the primary contributors to mortality in septic patients [2]. Sepsis often follows major surgeries, severe burns, or trauma and is associated with high morbidity, mortality, and economic costs [3]. Recently, the mortality rate of sepsis has been progressively decreasing despite its increasing incidence rate. This can be attributed to advancements in intensive care, revised sepsis guidelines, and a greater understanding of the importance of early detection and prompt intervention [4]. However, sepsis continues to pose a major risk to human health and remains a global issue that requires attention [3]. The prompt identification and standardized management of sepsis are imperative due to its time-sensitive condition. The lungs are particularly vulnerable to damage during the early stages of sepsis [5]. Over 50% of patients with ALI due to sepsis face high mortality within 48–72 h of treatment [5]. Effective management of ALI requires early diagnosis, adequate resuscitation, and timely care.

Multiple studies have shown that novel immunological markers can be used as prognostic indicators and therapeutic targets in sepsis. Circulating mRNA levels in the blood of septic patients have shown variations, indicating their potential as diagnostic biomarkers [6–8]. The circulating blood can serve as an indicator of the pulmonary circulation and the overall condition of the lungs. Gene expression similarities between blood and lung tissue can be investigated due to shared biological processes. Exosomes, platelets, and leukocytes, found in whole blood, have a considerable effect on the formation and progression of diseases [9]. Therefore, peripheral whole-blood mRNAs may offer novel strategies for addressing sepsis-induced ALI.

High-throughput (HTP) sequencing is a powerful tool for identifying disease-related genes and exploring diagnostic and therapeutic strategies by analyzing gene expression variations. Machine learning algorithms have demonstrated a significant potential in understanding the fundamental connections within high-dimensional datasets using supervised or unsupervised methods [10–12]. Furthermore, machine learning facilitates the analysis of high-dimensional transcriptome data and the identification of biologically significant genes [13, 14].

In contrast to previous studies, the current analysis involved integrating various HTP sequencing data of sepsis collected over 24 h. Furthermore, machine learning was employed to identify distinctive genes in sepsis for the first time. Immune cell (IC) infiltration is the process by which ICs migrate to disease tissues. Moreover, the study examined the associations between signatures and ICs to gain insights into the immunological and molecular processes underlying sepsis progression. This analysis aimed to improve understanding of immunological pathways involved in ALI progression.

## 2. Materials and Methods

### 2.1. Data Acquisition and Processing

**Figure 1:**
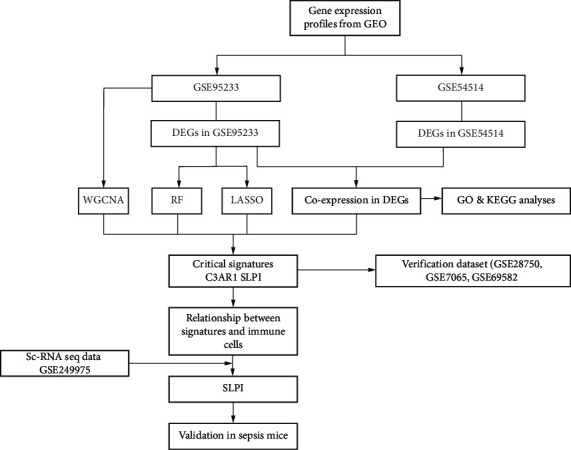
Flowchart of the bioinformation.

An overview of the data analysis process is shown in Figure 1. The initial microarray datasets GSE95233 and GSE54514 were obtained from the public repository for microarray data, the National Center for Biotechnology Information-GEO (NCBI-GEO). The GSE95233 dataset includes 22 healthy participants and 29 patients who developed septic shock within 24 h (Table 1), based on the GPL570 platform. Based on the GPL6947 platform, GSE54514 includes samples from 18 healthy participants and 35 patients with septic shock within 24 h. The annotation files for GPL570 and GPL54514 were obtained from GEO. The relative expressions of all probe IDs were determined to represent the expression level of a single gene when multiple probe IDs were measured. Tables 1 and 2 provides a summary of the clinical data associated with these datasets. Furthermore, the sets GSE28750, GSE57065, and GSE69528 were employed to validate the expression of signatures. Subsets of spleen-specific IC responses to sepsis were identified using scRNA-seq data from GSE249975 to capture the full spectrum of IC responses. The Bioconductor package in R was used for quality control (QC) and microarray data preprocessing, including background noise correction and normalization.

### 2.2. Detection of DEGs

In this study, differential expression analysis was carried out to evaluate DEGs using the Bioconductor package limma (V3.46.0) [15]. The *p* values were adjusted *via* Benjamini–Hochberg's false discovery rate (FDR). Genes with an adjusted *p* value ≤ 0.05 and |Log2 fold − change(log2 FC)| ≥ 0.585 were considered DEGs. Volcano plots were plotted using the ggplot2 V3.3.5 package in R software. Heatmaps for DEGs from each dataset were drawn *via* the Pheatmap V1.0.12 in R software.

### 2.3. Detection of DEGs Via Functional Enrichment Analysis

Functional enrichment analysis was conducted *via* the clusterProfiler V3.18.1 and the Goplot V1.0.2 packages to investigate the effect and pathways associated with overlapped DEGs. The significance threshold for this analysis was set at *p* ≤ 0.05 and *q* ≤ 0.05. An enrichment analysis was carried out, and the results were shown for all overlapped DEGs using Gene Ontology (GO) (biological processes (BP), cellular component (CC), and molecular function (MF)), and Kyoto Encyclopedia of Genes and Genomes (KEGG) pathways. Further, GO enrichment analysis was conducted to evaluate the overlapped increased or decreased levels of DEGs.

### 2.4. Critical Gene Signature Screening and Verification

In this study, different algorithms were employed to evaluate new and significant indicators for sepsis. These algorithms included random forests (RFs), weighted gene coexpression network analysis (WGCNA), and least absolute shrinkage and selection operator (LASSO) logistic regression. The RF model was constructed using the randomForest V4.7–1 package in R. LASSO logistic regression analysis was performed using the glmnet V4.1–4 package. The WGCNA was conducted using the WGCNA V1.71 package. Overlapping genes across all classification models were identified for further analysis. Gene expression differences between the sepsis and control groups were analyzed using validation datasets (GSE28750, GSE57065, and GSE69528) to evaluate the accuracy of key biomarkers.

### 2.5. Determination, Examination, and Correlation of Infiltrated ICs

CIBERSORT was used to evaluate the infiltration of 22 distinct IC types. The corrplot V0.92 package in R was used to construct a heat map showing correlations between infiltrated ICs, with a significance threshold of *p* ≤ 0.05. The heat map illustrated differences in IC infiltration between the control and sepsis groups. The Wilcoxon rank sum test was then used to assess the different infiltrated ICs between both groups. Lastly, the Spearman correlations between infiltrating ICs and biomarkers were analyzed. The results were visualized *via* the ggstatsplot V0.9.1 package in R.

### 2.6. Single-Cell RNA Sequencing Data Analysis

The GEO database was searched for the identifiers, gene characteristics, and gene count matrix data of GSE249975 that had been preprocessed by Cellranger (10x Genomics). After importation into R, these data were analyzed *via* the Seurat V4.1.0 tool [16]. Firstly, the QC process involved the exclusion of cells that met the following criteria: a gene count/cell ranging from 200 to ≤ 2500 and a proportion of mitochondrial genes exceeding 5%. Data were normalized *via* the normalized data function. The top 2000 DEGs were selected *via* the “vst” method in the FindVariableFeatures function to conduct the downstream analysis. Data were scaled using the ScaleData function and analyzed *via* PCA, cluster analysis, and uniform manifold approximation and projection (UMAP) dimensional reduction with RunPCA, FindClusters, and RunUMAP functions. After that, the cell clusters were observed by the UMAP plots generated by the DimPlot function. Different expressions of signatures were identified *via* the FindAllMarkers function. The VlnPlot function was employed to generate violin plots. Moreover, cell types were annotated using the R software SingleR V1.4.1 package [17]. HumanPrimaryCellAtlasData reference was obtained from the celldex V1.0.0 package.

### 2.7. Animal Model Development

All animal-related protocols adhered to the guidelines outlined in the Guide for the Care and Use of Laboratory Animals. The current study was approved by the Animal Protection and Use Committee of Suzhou Hospital of Integrated Traditional Chinese and Western Medicine (approval no. 2024002). Male wild-type C57BL/6 mice, aged 6–8 weeks, were used for the study. The polymicrobial septic model was established using cecal ligation and puncture (CLP). Mice were anesthetized with 2% isoflurane inhalation, and a midline abdominal incision was made. The cecum was ligated approximately 1 cm proximal to its distal end using a 4–0 silk suture and punctured twice with a 22-gauge needle to create two through-and-through holes. For the sham group, laparotomy was performed without CLP. To prevent dehydration from surgery, 1 mL of normal saline was administered subcutaneously. Moreover, 0.05 mg/kg buprenorphine was injected subcutaneously for analgesia. The right upper lobe of the lungs was harvested 24 h postsurgery, and tissue samples were collected from five mice per group. Peripheral blood was collected from each mouse. Plasma was separated and stored at −80°C for later use.

### 2.8. Lung Wet/Dry Weight Ratio

Wet weight was recorded after resecting the left lobe of the lung. These lungs were desiccated at 60°C for 72 h, after which they were weighed to determine their dry weight. The ratio of wet to dry weight was measured to examine the lung edema.

### 2.9. Lung Injury Score

Lung injury was assessed using a semiquantitative scoring system as described previously [18]. Four pathological parameters were scored on a scale of 0–4: alveolar congestion, hemorrhage, leukocyte infiltration or neutrophil aggregation in airspaces or vessel walls, and alveolar wall thickness. The total score for all parameters was used for comparative analysis.

### 2.10. Real-Time qRT-PCR Validation

RNA was extracted from lung tissues using TRIzol reagent (Pufei Biological, USA). Complementary DNA (cDNA) was synthesized using a reverse transcription kit (Vazyme, Nanjing, China) according to the manufacturer's instructions. GAPDH was used as an internal control for comparative gene expression analysis. Gene expression levels were quantified using the 2^−*ΔΔ*Ct^ method (*n* = 4).  Table 3 provides the primer sequence of SLPI and GAPDH.

### 2.11. Western Blotting

Lung tissue samples were lysed using RIPA lysis buffer (Servicebio, Wuhan) containing 1% phenylmethanesulfonyl fluoride (PMSF, Servicebio). The extracted proteins were separated on 10%–15% SDS-PAGE and transferred onto polyvinylidene fluoride (PVDF) membranes. Membranes were blocked with 5% skim milk to prevent nonspecific binding and incubated with primary antibodies at 4°C for 12 h. Secondary antibodies, including anti-rabbit-HRP and anti-mouse-HRP (1:3000; Proteintech), were applied at 37°C for 2 h. *β*-Actin served as the internal control. Protein bands were visualized using an enhanced chemiluminescence (ECL) detection kit (Bio-Rad, USA).

### 2.12. Enzyme-Linked Immunosorbent Assay

Tissue samples were harvested under stereoscopic observation. Tissue and plasma homogenized in separation buffer with protease inhibitors, and centrifuged at 13,000 g for 30 min at 4°C. The supernatant was analyzed using a mouse-specific SLPI ELISA kit (R&D, DY1735-05), C3AR1 (Biocompare, abx392273) according to the manufacturer's instructions. Approximately 100 *μ*L of each sample and calibrators were added to wells coated with cytokine capture antibodies. After 2 h of incubation, primary antibodies were added for 2 h, followed by secondary antibodies for 1 h. The reaction was detected using an MSD reagent, and plates were read on a MesoQuickPlex SQ 120 system.

### 2.13. Hematoxylin and Eosin (H&E) Staining

Longitudinal sections of lung tissue were stained with H&E to evaluate the dimensions of lung injury cavities (*n* = 3/group). Sections were deparaffinized, rehydrated using a graded alcohol series, and stained according to the manufacturer's protocol. Three lung sections spaced 50 *μ*m apart were examined per animal. Observations were made using an Olympus microscope with a 40x objective lens. Cavity dimensions were quantified using ImageJ software, and cavity size percentage was calculated using the respective formula:

 $$\begin{array}{c} \text{Cavity size percentage}=\text{Cavity size}\left(\mu \text{m}\right)/\text{Total area of the section}\left(\mu \text{m}\right)\times 100. \end{array}$$

### 2.14. Immunofluorescence

Immunofluorescence staining was performed as described previously [19]. Paraffin-embedded tissue sections were dewaxed, hydrated, and subjected to antigen retrieval. Sections were incubated with SLPI primary antibody (1:50, R&D, NBP1-76803) for 24 h at 4°C in the dark, followed by incubation with goat anti-rabbit secondary antibody (1:1000, Santa Cruz Biotechnology, sc-516102) for 1 h. After DAPI staining, tissues were visualized using a confocal laser microscope (LEICA, Germany). Quantification of cells was performed for all mice (*n* = 3/group).

### 2.15. Statistical Analysis

Data were analyzed using GraphPad Prism version 8.0. A one-way ANOVA test was applied to evaluate differences between the histological and molecular groups. Results were expressed as means ± standard deviation (SD), with statistical significance at *p* ≤ 0.05.

## 3. Results

### 3.1. Detection of DEGs

**Figure 2:**
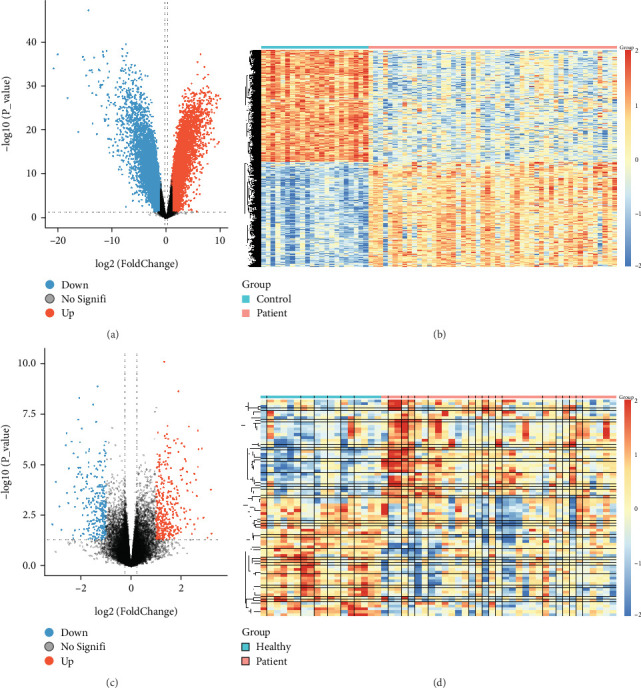
Volcano plot and heat map. (a) Volcano plots of DEG distribution in GSE95233. (b) Heatmaps of DEGs in GSE95233. (c) Volcano plots of DEG distribution in GSE54514. (d) Heatmaps of DEGs in GSE54514.

Differential expression analysis identified 184 DEGs in GSE54514, including 103 upregulated and 81 downregulated genes (Figure 2a,b). Moreover, 944 DEGs were identified in GSE95233, comprising 703 upregulated and 448 downregulated genes. The distribution of these DEGs was visualized using volcano plots (Figure 2c,d).

### 3.2. GO and KEGG Pathway Analyses of DEGs

**Figure 3:**
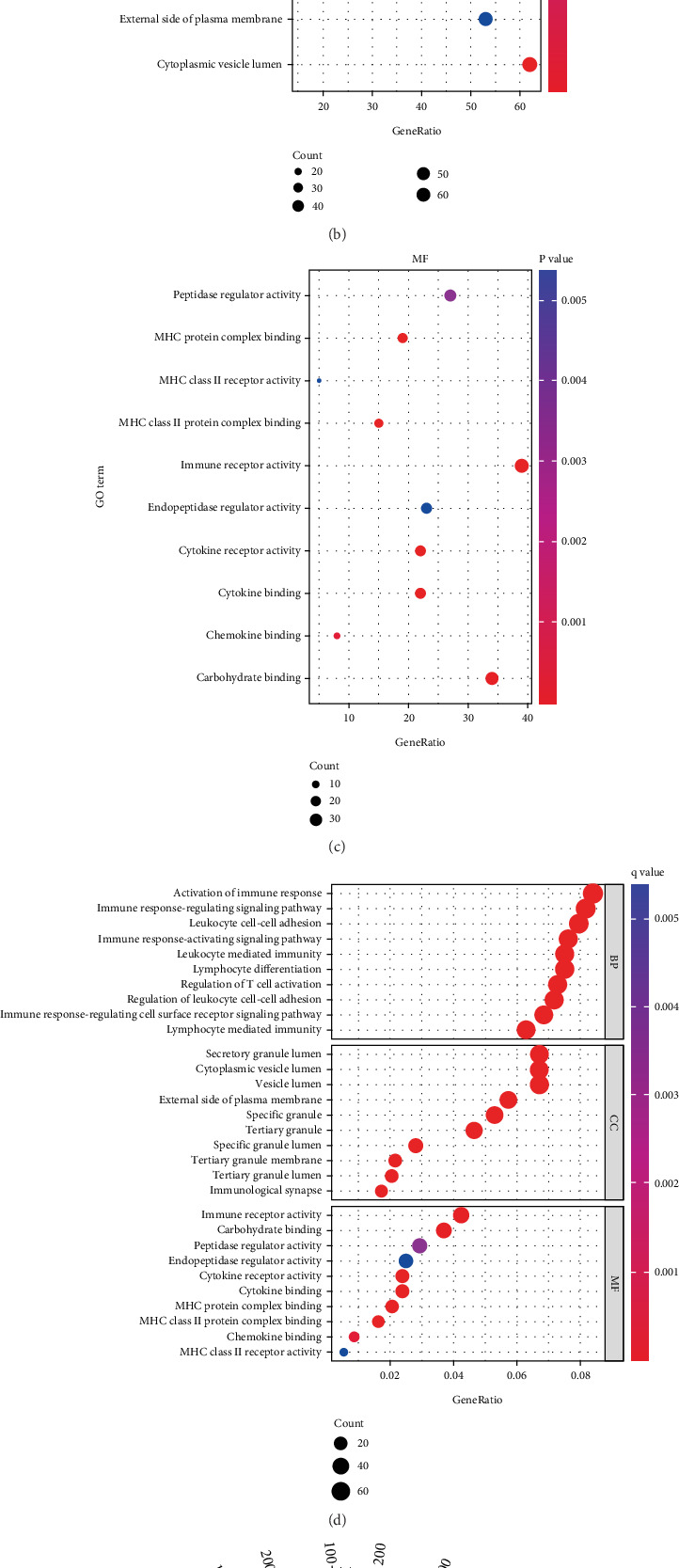
Gene Ontology (GO) and Kyoto Encyclopedia of Genes and Genomes (KEGG) pathway enrichment analyses of differentially expressed genes (DEGs). (a–d) Bubble charts show GO-enriched items of DEGs in three functional groups: biological processes (BP), cell composition (CC), and molecular function (MF). The *x*-axis labels represent gene ratios, and *y*-axis labels represent GO terms. The size of circle represents gene count. Different colors of circles represent different adjusted *p* values. (e) Circle plot shows KEGG-enriched items of DEGs. The height of the bar in the inner ring indicates the significance of the term, and color corresponds to the *p* value.

Functional enrichment analyses were conducted using GO and KEGG pathways for overlapping DEGs. GO analysis revealed significant enrichment in BP related to neutrophil activity (activation and degradation), immune responses, and T cell activation. The top five enriched terms in the CC domain were specific granule, secretory granule lumen, cytoplasmic vesicle lumen, and tertiary granule. The most enriched terms within the MF category included immune receptor activity, MHC class II protein complex binding, cytokine receptor activity, cytokine binding, and carbohydrate interactions (Figures 3a, 3b, and 3c). The most significantly enriched GO terms across BP, CC, and MF categories are illustrated in Figure 3d. KEGG pathway analysis identified the most abundant pathways as those related to Th17 cell differentiation, cytokine receptor interactions, chemokine signaling, B cell receptor signaling, and natural killer cell–mediated cytotoxicity (Figure 3e and Table 4).

### 3.3. Identification of Key Signatures

**Figure 4:**
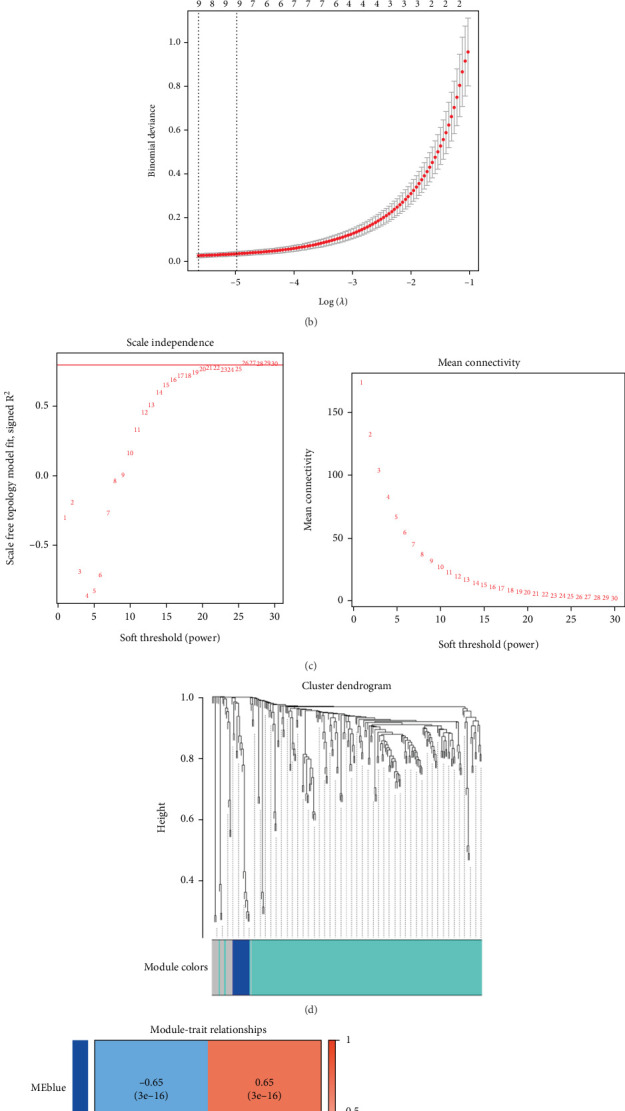
Screening of critical signatures via multiple machine learning. (a, b) Establishment of signatures by least absolute shrinkage and selection operator (LASSO) logistic regression analysis. LASSO coefficient profile of the 9 genes, and different colors represent different genes. Selection of the optimal parameter (lambda) in the LASSO model and generation of a coefficient profile plot. (c) Process of weighted gene coexpression network analysis (WGCNA). Analysis of network topology for various softthresholding powers. The *x*-axis reflects the soft-thresholding power. The *y*-axis reflects the scale-free topology model fit index and the mean connectivity. (d) Clustering dendrogram of differentially expressed genes related to sepsis, with dissimilarity based on topological overlap, together with assigned module colors. (e) Module–trait associations. Each row corresponds to a module, and each column corresponds to a trait. Each cell contains the corresponding correlation and *p* value. The table is color-coded by correlation according to the color legend. (f) Venn diagram shows the intersection of critical signatures obtained by the three strategies.

Given the more prominent differential expression observed in GSE95233 compared to GSE54514, subsequent analyses were exclusively performed using GSE95233. Three computational approaches were employed to identify critical markers: RF algorithm, LASSO logistic regression, and WGCNA. The RF algorithm identified 206 candidate genes, while the LASSO logistic regression technique highlighted nine genes (Figure 4a,b). Using default parameters, WGCNA identified three significant coexpression modules (Figures 4c, 4d, and 4e). Module–trait correlation analysis revealed several modules associated with sepsis. Integrating the results of the three algorithms, C3AR1 and SLPI were identified as overlapping upregulated genes, as depicted in the Venn diagrams (Figure 4f).

### 3.4. Confirmation of C3AR1 and SLPI in Sepsis

**Figure 5:**
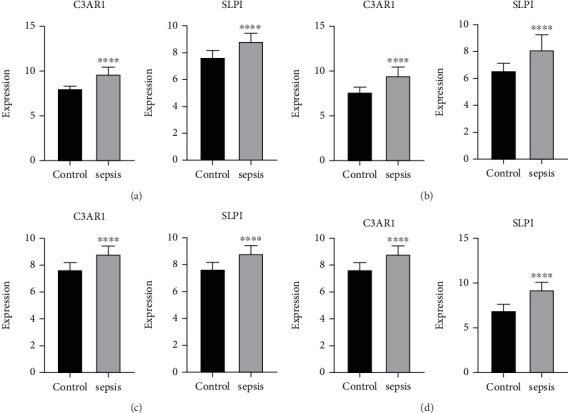
The expressions of C3AR1 and SLPI in (a) GSE28750, (b) GSE7065, (c) GSE69528, and (d) GSE95233.

The expression levels of C3AR1 and SLPI were examined in sepsis datasets. Both genes were significantly upregulated in sepsis compared to control groups in the GSE28750 and GSE7065 datasets (all *p* < 0.01). Similarly, in the validation datasets GSE69528 and GSE95233, the relative expression of C3AR1 and SLPI in advanced sepsis was significantly higher than in the control group (Figures 5a, 5b, 5c, and 5d).

### 3.5. Analysis of Infiltrated ICs

**Figure 6:**
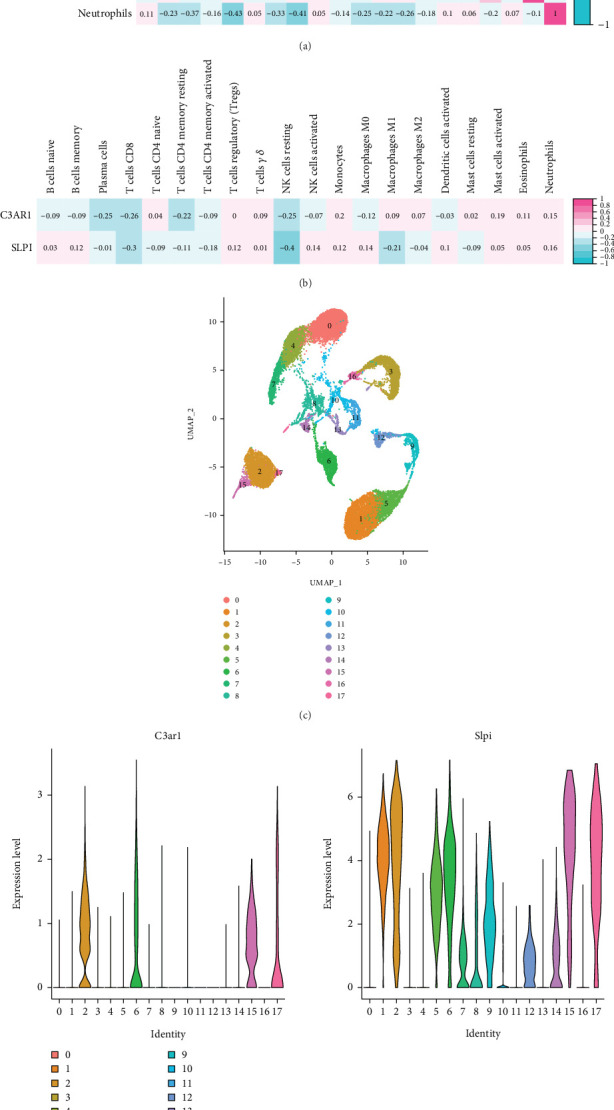
Immune cell infiltration analysis and relationships between key signatures and immune cells in sepsis. (a) Heatmap of correlation in 17 types of immune cells. The size of the colored squares represents the strength of the correlation; red represents a positive correlation, and blue represents a negative correlation. Darker color implies stronger association. (b) Correlations between C3AR1, SLPI, and infiltrating immune cells. (c) UMAP visualization of clustering revealing 17 cell clusters. (d) Violin plots show expression distribution of C3AR1 and SLPI mRNA in different cell clusters in the spleen. Cluster identities: 0, T cells; 1, T cells; 2, endothelial cells; 3, smooth muscle cells; 4, macrophage; 5, monocyte; 6, chondrocytes; 7, monocyte; 8, B cell; 9, endothelial cells; 10, tissue stem cells; 11, smooth muscle cells; 12, B cell; 13, monocyte; 14, NK cell; 15, NA. (e) Distribution of C3AR1 and SLPI in immune cells.

Clinical and experimental evidence increasingly suggests that immune processes play a critical role in sepsis progression [20, 21]. Understanding the relationship between key markers and IC infiltration during the early stages of sepsis is crucial. Using the CIBERSORT algorithm, 22 IC phenotypes were analyzed in the GSE249975 dataset. Five types of ICs with very low abundance were excluded, leaving 17 types of ICs for analysis. The correlation heat map revealed significant negative correlations between specific pairs of ICs, including memory B cells and plasma cells, monocytes and macrophages (M0), CD4 memory resting cells and macrophages (M0), and resting and activated mast cells (Figure 6a,b). Significant positive correlations were observed between resting mast cells, CD4 memory cells, and macrophages (M1), as well as between resting T cells, CD4 memory cells, and macrophages (M2).

### 3.6. Gene Levels of C3AR1 and SLPI by scRNA-seq Analysis

A scRNA-seq database was employed to investigate the expression of key signatures within ICs of the spleen during sepsis. After QC and cell purification, gene expression levels of C3AR1 and SLPI were analyzed to generate unbiased cell clusters. Clustering identified 17 distinct subpopulations, visualized using a UMAP plot. In the spleen tissue from sepsis models, SLPI mRNA expression was significantly elevated in macrophages (cluster #4), NK cells (cluster #14), T cells (cluster #1), endothelial cells (clusters #2 and #9), monocytes (cluster #5), chondrocytes (cluster #6), and B cells (clusters #8 and #12) (*p* ≤ 0.001) (Figures 6c, 6d, and 6e).

### 3.7. Expression of SLPI in Sepsis ALI

**Figure 7:**
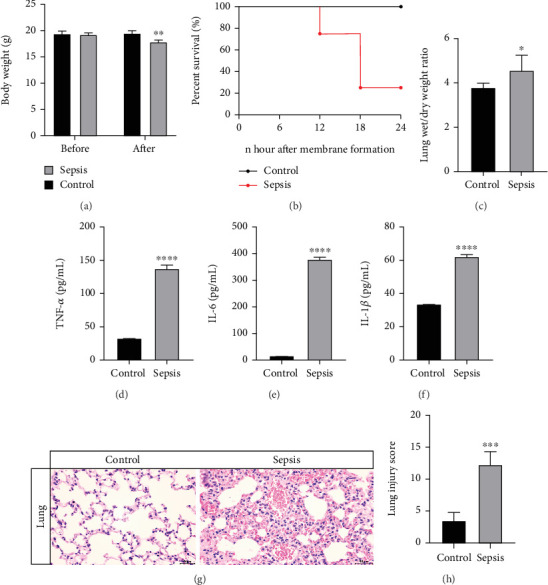
CLP-induced lung pathological changes in mice. (a) Body weight in mice between the control and sepsis groups. (b) Survivals in mice between the control and sepsis groups. (c) W/D ratios in mice lung tissue. Lung wet/day ratios were measured at 24 h. (d–f) TNF-*α*, IL-6, and IL-1*β* expressions in mice between the control and sepsis groups. (g) Lung tissues were collected 24 h after the operation and examined by light microscopy after H&E staining. (h) Lung damage was evaluated using the lung scores described in the methods.

**Figure 8:**
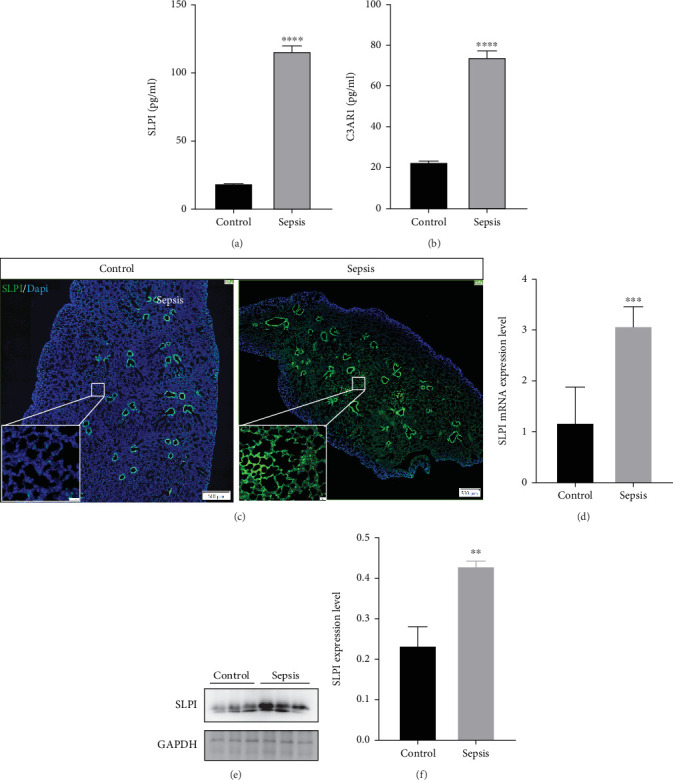
Expression of SLPI in mice lung tissues. (a, b) Plasma SLPI and C3AR1 expressions in mice between the control and sepsis groups. (c) Immunostaining for SLPI in lung tissues. (d) SLPI mRNA in mice between the control and sepsis groups. (e, f) Protein levels of SLPI in mouse lung and were assessed by Western blot analysis.

An ALI mouse model was established to investigate the expression of SLPI. Mice in the sepsis group exhibited significant weight loss compared to the healthy control group (Figure 7a). Six out of eight mice died due to sepsis within 24 h (Figure 7b). The lung wet-to-dry weight ratio, an indicator of pulmonary edema, was significantly elevated in the sepsis group (Figure 7c). Similarly, mRNA levels of proinflammatory mediators, including *TNF-α*, *IL-1β*, and *IL-6*, were upregulated in the lung tissue following sepsis (Figures 7d, 7e, and 7f). These findings are consistent with prior reports indicating that the severity of lung injury is most pronounced 24 h postsepsis [22]. Lung tissues were harvested 24 h after surgery for further analysis. H&E staining revealed significant histological changes in the lungs of CLP-induced septic mice, including focal congestion, swelling, thickened alveolar septa infiltrated with inflammatory cells, and collapsed or overdilated alveoli (Figure 7g). The lung injury scores in septic mice were significantly elevated, reflecting sepsis-induced ALI (Figure 7h). As expected, CLP treatment induced SLPI, and C3AR1 significantly increased in the plasma (Figure 8a,b). Furthermore, immunofluorescence analysis demonstrated the overexpression of SLPI in the lung tissue during sepsis (Figure 8c). Western blot analysis further confirmed that SLPI protein levels were elevated in the lungs of septic mice subjected to CLP (Figure 8e,f). SLPI mRNA levels were approximately threefold higher than those observed in sham-operated controls (Figure 8d).

## 4. Discussion

Sepsis is a syndrome characterized by a dysregulated response of the body to invading pathogens, leading to severe systemic inflammation and compromised function of multiple vital organs [23]. Among affected organs, the lungs are the most commonly impaired, with acute respiratory distress syndrome (ARDS)—clinically referred to as ALI—serving as a key predictor of mortality in septic patients [24].

Current medical guidelines for sepsis emphasize the critical importance of early detection and timely administration of antibiotics to improve patient outcomes [25]. To improve the survival of patients with ALI, identifying specific target biomarkers and understanding the patterns of IC infiltration associated with septic ICs are essential. These efforts can significantly advance knowledge of the immunological effects of sepsis. This study aimed to identify a sepsis-specific biomarker within 24 h of onset and to evaluate its impact on ALI.

Machine learning analyses suggest that correlations between fluid proteomes and single-cell transcriptomes can accurately predict patterns of lung function changes across different methods. This study focused on identifying specific blood biomarkers for sepsis and examining the effect of lung injury on sepsis outcomes. Previous studies have shown that the majority of sepsis-related deaths result from untreated opportunistic infections and compromised immune systems [26, 27]. Sepsis represents a complex interplay between host immune responses and infections, with pathogens capable of disrupting various aspects of host immunity [28]. Moreover, sepsis significantly affects immune functionality by altering the development, maturation, and survival of ICs, as well as their ability to respond effectively [29].

In this study, integrative bioinformatics analysis was used to identify 1150 DEGs in the GSE95233 dataset (703 upregulated and 447 downregulated) between controls and sepsis at 24 h. Moreover, 611 overlapping DEGs were identified in the GSE54514 dataset, with 361 upregulated and 250 downregulated. GO enrichment analysis revealed that these overlapping DEGs were primarily associated with immune response pathways, including immune response modulation, immune response stimulation, positive control of cytokine recruitment, leukocyte-mediated immune responses, T cell activation, and myeloid leukocyte activation. However, KEGG pathway analysis highlighted associations with blood cell lineage, *Staphylococcus aureus* infection, and differentiation of Th1, Th2, and Th17 cells. These findings align with existing evidence associating sepsis with inflammation and immunity.

Three machine learning algorithms—RF, LASSO logistic regression, and WGCNA—were applied to classify sepsis-specific signature markers [30, 31]. WGCNA is a machine learning technique widely used for feature ranking and identifying key features for classification [32]. The RF model, a nonparametric supervised approach, was incorporated into a decision tree algorithm developed using segmented datasets. Training and RF-based classification model analysis were conducted to differentiate sepsis from control samples [33]. LASSO logistic regression, another machine learning method, identifies features by minimizing classification error [34]. Integrating the results of these approaches, C3AR1 and SLPI were identified as key sepsis-associated genes. Verification analyses confirmed their accuracy, demonstrating the effectiveness of this integrated prediction approach. Considerable evidence supports a strong association between C3AR1 and SLPI and sepsis, highlighting their significance in sepsis pathophysiology [35, 36].

Given the critical role of immunity during the early stages of sepsis, the relationship between IC signatures and sepsis-associated gene markers was further examined. Findings revealed varying levels of correlation between C3AR1 and SLPI and ICs. Single-cell omics analysis demonstrated significantly increased SLPI mRNA expression in ICs during sepsis, whereas C3AR1 expression did not show significant differences in sepsis-affected ICs. These results were further validated in a sepsis-induced ALI mouse model. This study identifies SLPI as a novel biomarker for early-stage sepsis. Unlike dendritic cells, macrophages, and neutrophils, SLPI is predominantly secreted by epithelial cells [37]. Anti-inflammatory stimuli regulate the synthesis and release of SLPI, which increases during conditions such as pneumonia and sepsis to counteract tissue injury and regulate inflammation [38]. An association exists between elevated plasma SLPI levels and the degree of organ dysfunction in sepsis patients. Previous studies have shown that males with community-acquired pneumonia (CAP) show higher plasma SLPI levels [39]. This study confirmed that SLPI levels are significantly elevated in sepsis-induced ALI, further supporting its potential as a biomarker.

SLPI, a 12-kDa nonglycosylated cationic protein, has emerged as an important regulator of innate and adaptive immunity and a key component in tissue regenerative programs. SLPI is broadly expressed at both mRNA and protein levels in epithelial cells, including those lining the respiratory, digestive, and reproductive tracts, as well as in the parotid glands, breast, kidney, and skin [40]. Its most well-documented role is the reversible inhibition of neutrophil elastase (NE), accounting for 80%–97% of the NE inhibitory capacity in human upper respiratory tract secretions [38]. As a regulator of enzymatic activity, SLPI controls inflammatory responses by attenuating monocyte/macrophage responses to lipopolysaccharide (LPS) through the inhibition of the transcription factor NF-*κ*B [41]. Beyond protease activity, SLPI has demonstrated the ability to inhibit bacterial and fungal growth and control viral infections [41, 42]. SLPI-mediated control protects the host from excessive or dysregulated inflammation seen in infectious, allergic, and autoinflammatory diseases while supporting healing through its effects on cell proliferation, differentiation, and apoptosis [38].

Studies investigating SLPI in the context of sepsis are limited, and SLPI production over the course of sepsis, or in relation to clinical characteristics and etiology has not been studied [36]. SLPI can inhibit the inflammatory response and apoptosis of HK2 cells induced by LPS, which may be involved in the protective mechanism of renal tubular cells in the response to sepsis, and is a potential target for the treatment of sepsis-associated acute kidney injury [43]. SLPI KO mice had a higher susceptibility to acute *P. aeruginosa* infection in comparison to their wild-type counterparts. At 24 h postinfection, 83% of wild-type mice had survived the infection, which suggest that endogenous SLPI is involved in controlling the inflammatory responseto protect the host [44].

Previously, C3AR1 was thought to function solely in the innate immune response as part of the complement cascade. However, its roles have since been expanded to include cancer progression, neurogenesis, and pituitary hormone release [45]. The current study demonstrated that low C3AR1 expression was positively associated with activated ICs, including B cells, CD4^+^ T cells, CD8^+^ T cells, and NK cells, suggesting that reduced C3AR1 expression contributes to an immune-activated microenvironment. Therefore, C3AR1 may affect coagulation cascade dysregulation by modulating IC infiltration. The expression level of C3AR1 efficiently reflects the immune microenvironment during sepsis, offering potential guidance for immune-modulating agents to achieve immune homeostasis [35]. Furthermore, C3AR1 may play a role in the differentiation between the M1 and M2 macrophages [46]. This differentiation can shift macrophages toward an M2 phenotype, where excessive activation results in elevated proinflammatory cytokine production in the early stages of sepsis. If this proinflammatory response is not properly regulated, it can lead to a cytokine storm, macrophage apoptosis, and, ultimately, immunosuppression [47]. Thus, C3AR1 appears to play a critical role in sepsis pathophysiology and may serve as a prognostic marker or therapeutic target.

This study concludes that SLPI serves as a reliable biomarker for the early stages of sepsis and sepsis-induced ALI. Furthermore, the findings highlight a significant role of ICs in the initiation and progression of sepsis during its early stages. SLPI showed considerable associations with various IC types, suggesting that these cells play a significant role in sepsis progression. Detailed analysis of these ICs could aid in identifying potential targets for immunotherapy and improving immunomodulatory interventions for septic patients.

## Figures and Tables

**Table 1:** Descriptive data of the mRNA study groups in GSE95233.

	**Patients (** **n** = 29**)**	**Health control (** **n** = 22**)**	**p** ** value**
Ages (years)	57.13 ± 6.98	59.16 ± 8.30	0.22
Male/female ratio	11/18	10/12	0.47
Time of sample collection	Day 1		

**Table 2:** Descriptive data of the mRNA study groups in GSE54514.

	**Patients (** **n** = 35**)**	**Health control (** **n** = 18**)**	**p** ** value**
Ages (years)	50.6 ± 4.35	54.78 ± 5.33	0.14
Male/female ratio	21/15	8/10	0.86
Time of sample collection	Day 1		

**Table 3:** Primer sequence of gene.

**Gene**	**Forward primer**	**Reverse primer**
SLPI	AGAAAGACACTTGCCCAGGA	CCTGAACCCTACTCCAAGCA
GAPDH	GATGGTGATGGGTTTCCCGT	AGTGCCAGCCTCGTCTCATA

**Table 4:** KEGG-enriched items of DEGs.

**ID**	**Description**	**GeneRatio**	**p** ** value**	**p** **.adjust**	**q** ** value**	**Count**
hsa04659	Th17 cell differentiation	37/515	2.78*e* − 19	8.83*e* − 17	7.28*e* − 17	37
hsa04640	Hematopoietic cell lineage	34/515	7.53*e* − 18	1.20*e* − 15	9.87*e* − 16	34
hsa04658	Th1 and Th2 cell differentiation	32/515	4.69*e* − 17	4.97*e* − 15	4.10*e* − 15	32
hsa05321	Inflammatory bowel disease	22/515	7.65*e* − 12	6.08*e* − 10	5.01*e* − 10	22
hsa05145	Toxoplasmosis	27/515	1.83*e* − 10	1.02*e* − 08	8.39*e* − 09	27
hsa05235	PD-L1 expression and PD-1 checkpoint pathway in cancer	24/515	1.92*e* − 10	1.02*e* − 08	8.39*e* − 09	24
hsa05310	Asthma	13/515	7.67*e* − 09	3.48*e* − 07	2.87*e* − 07	13
hsa04672	Intestinal immune network for IgA production	16/515	1.04*e* − 08	4.14*e* − 07	3.41*e* − 07	16
hsa05330	Allograft rejection	14/515	1.49*e* − 08	5.24*e* − 07	4.32*e* − 07	14
hsa04612	Antigen processing and presentation	20/515	1.65*e* − 08	5.24*e* − 07	4.32*e* − 07	20
hsa04660	T cell receptor signaling pathway	25/515	3.12*e* − 08	9.03*e* − 07	7.44*e* − 07	25
hsa05332	Graft-versus-host disease	14/515	6.53*e* − 08	1.73*e* − 06	1.43*e* − 06	14
hsa05140	Leishmaniasis	19/515	7.39*e* − 08	1.81*e* − 06	1.49*e* − 06	19
hsa05320	Autoimmune thyroid disease	14/515	1.62*e* − 06	3.67*e* − 05	3.03*e* − 05	14
hsa05166	Human T cell leukemia virus 1 infection	32/515	2.47*e* − 06	5.23*e* − 05	4.31*e* − 05	32
hsa05169	Epstein–Barr virus infection	30/515	2.74*e* − 06	5.44*e* − 05	4.49*e* − 05	30
hsa04940	Type I diabetes mellitus	12/515	4.83*e* − 06	9.04*e* − 05	7.45*e* − 05	12
hsa05416	Viral myocarditis	15/515	6.52*e* − 06	0.000115169	9.49*e* − 05	15
hsa04514	Cell adhesion molecules	24/515	1.67*e* − 05	0.000279531	0.000230398	24
hsa05152	Tuberculosis	26/515	2.10*e* − 05	0.000333494	0.000274876	26
hsa05323	Rheumatoid arthritis	17/515	2.82*e* − 05	0.00042691	0.000351872	17
hsa04064	NF-kappa B signaling pathway	18/515	3.55*e* − 05	0.000513801	0.00042349	18
hsa05150	*Staphylococcus aureus* infection	17/515	4.31*e* − 05	0.000596384	0.000491558	17
hsa05340	Primary immunodeficiency	10/515	5.27*e* − 05	0.000698037	0.000575343	10
hsa05202	Transcriptional misregulation in cancer	26/515	7.10*e* − 05	0.000902043	0.000743491	26
hsa04061	Viral protein interaction with cytokine and cytokine receptor	17/515	7.38*e* − 05	0.000902043	0.000743491	17
hsa04650	Natural killer cell–mediated cytotoxicity	20/515	9.51*e* − 05	0.00112018	0.000923286	20
hsa04380	Osteoclast differentiation	20/515	0.000130723	0.001484637	0.001223683	20
hsa04060	Cytokine–cytokine receptor interaction	32/515	0.00075042	0.008228742	0.006782379	32
hsa00512	Mucin type O-glycan biosynthesis	8/515	0.001018198	0.0107929	0.008895836	8
hsa05164	Influenza A	21/515	0.001191443	0.012221898	0.010073659	21
hsa04621	NOD-like receptor signaling pathway	22/515	0.00150978	0.015003435	0.012366287	22
hsa05221	Acute myeloid leukemia	11/515	0.001819445	0.017532835	0.014451096	11
hsa05142	Chagas disease	14/515	0.002727186	0.025507208	0.021023816	14
hsa04662	B cell receptor signaling pathway	12/515	0.003842922	0.034233572	0.02821635	12
hsa04145	Phagosome	18/515	0.003875499	0.034233572	0.02821635	18
hsa00500	Starch and sucrose metabolism	7/515	0.004660146	0.040052069	0.033012132	7
hsa04062	Chemokine signaling pathway	21/515	0.004904958	0.041046755	0.033831982	21
hsa04210	Apoptosis	16/515	0.00668656	0.054521185	0.044938017	16
hsa05135	Yersinia infection	16/515	0.007169021	0.055603623	0.045830196	16
hsa05322	Systemic lupus erythematosus	16/515	0.007169021	0.055603623	0.045830196	16
hsa05170	Human immunodeficiency virus 1 infection	22/515	0.007495387	0.056750784	0.046775721	22

## Data Availability

The data that support the findings of this study are openly available in the GEO database at https://www.ncbi.nlm.nih.gov/mesh/.
